# Atypical Intracranial Epidermoid Cysts: Rare Anomalies with Unique Radiological Features

**DOI:** 10.1155/2015/528632

**Published:** 2015-01-15

**Authors:** Eric K. C. Law, Ryan K. L. Lee, Alex W. H. Ng, Deyond Y. W. Siu, Ho-Keung Ng

**Affiliations:** ^1^Department of Imaging & Interventional Radiology, Prince of Wales Hospital, The Chinese University of Hong Kong, Shatin, Hong Kong; ^2^Department of Radiology, Kwong Wah Hospital, Kowloon, Hong Kong; ^3^Department of Anatomical & Cellular Pathology, Prince of Wales Hospital, The Chinese University of Hong Kong, Shatin, Hong Kong

## Abstract

Epidermoid cysts are benign slow growing extra-axial tumours that insinuate between brain structures, while their occurrences in intra-axial or intradiploic locations are exceptionally rare. We present the clinical, imaging, and pathological findings in two patients with atypical epidermoid cysts. CT and MRI findings for the first case revealed an intraparenchymal epidermoid cyst that demonstrated no restricted diffusion. The second case demonstrated an aggressive epidermoid cyst that invaded into the intradiploic spaces, transverse sinus, and the calvarium. The timing of ectodermal tissue sequestration during fetal development may account for the occurrence of atypical epidermoid cysts.

## 1. Introduction

Epidermoid cysts are benign, slow growing extra-axial tumours that account for ~1% of all intracranial tumours [1]. Embryologically, they are derived from ectodermal inclusions during neural tube closure from the third to the fifth weeks of embryogenesis [1, 2]. They frequently occur at the cerebellopontine angles and parasellar regions, insinuating between brain structures. Conversely, epidermoid cysts in intraparenchymal or intradiploic locations are very rare, accounting for less than 5% of all intracranial epidermoid cysts [3]. Here, we report two cases of atypical epidermoid cysts; the first case demonstrated an intraparenchymal epidermoid cyst while the second case showed an epidermoid cyst that invaded into the intradiploic space and surrounding structures. Comparison on the salient radiological features of typical and atypical epidermoid cysts is emphasized.

## 2. Patient 1

**Figure 1:**
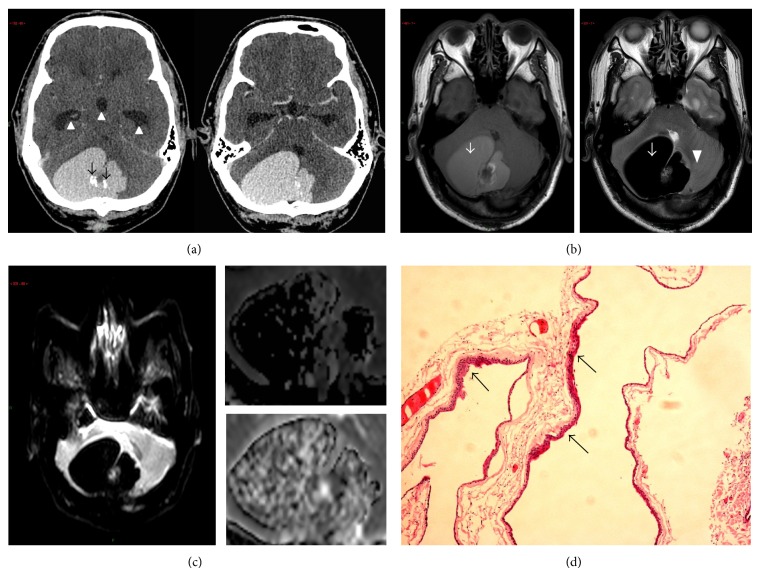
(a) Plain CT brain shows a lobulated, uniform hyperdense cystic lesion in right cerebellar hemisphere with coarse calcifications (black arrows) and no appreciable contrast enhancement (not shown). Note the acute angle the lesion makes with the calvarium and lobulated contour suggestive of its intraparenchymal location. Hydrocephalus involves the third ventricle and temporal horns of both lateral ventricles (white arrowheads) are due to the mass effect on the fourth ventricle. Histology confirmed the lesion as an epidermoid cyst. (b) T1-weighted (left) and T2-weighted (right) MRI images of the epidermoid cyst show mild T1 hyperintense and dramatic T2 hypointense signal (white arrows). The signal combination is completely opposite to the typical epidermoid cyst. There is no appreciable contrast enhancement after IV gadolinium (not shown). Note the lack of significant perilesional oedema in the cerebellum (arrowhead), a cardinal characteristic of an epidermoid cyst. (c) Diffusion weighted image (DWI) with *b* factor 1000 (left panel), magnified masked apparent diffusion coefficient (ADC) (right upper), and unmasked ADC (right lower) images demonstrate no appreciable restricted diffusion with marked hypointensity on the DWI. While the hypointense signal in the masked ADC map may suggest restricted diffusion, this was a result of postimage processing of ignoring low intensity voxels of bones or air. Raw unmasked ADC map (right lower panel) confirms the lack of restricted diffusion, as evident by its isointense signal. (d) H&E section (×100 magnification) of the postsurgical specimen shows thin cyst wall (black arrows) with keratinizing squamous epithelium; features are compatible with an epidermoid cyst.

A 47-year-old male with good past health was admitted for a one-month history of ataxia and headache. Plain CT brain (Figure 1(a)) showed an intra-axial hyperdense mass in the right cerebellar hemisphere with coarse calcific foci. The lesion was homogenous in appearance with lobulated margin with no appreciable enhancement after IV contrast (Figure 1(a)). Despite its size and mass effect (causing obstructive hydrocephalus), no significant vasogenic oedema was evident. On MRI, the lesion appeared markedly T2W hypointense and T1W hyperintense (Figure 1(b)), suggestive of either high proteinaceous content or subacute blood. No fluid restriction was demonstrated in the diffusion weighted images (DWI) and corresponding apparent diffusion coefficient (ADC) images (Figure 1(c)). Intraoperative findings showed extremely thick gelatinous substance, and histological examination of the surgical specimen showed cyst lining made of attenuated squamous epithelium and dystrophic calcification along with keratinous debris, findings compatible with an epidermoid cyst (Figure 1(d)). The patient recovered well following surgery and had no neurological sequelae.

## 3. Patient 2

**Figure 2:**
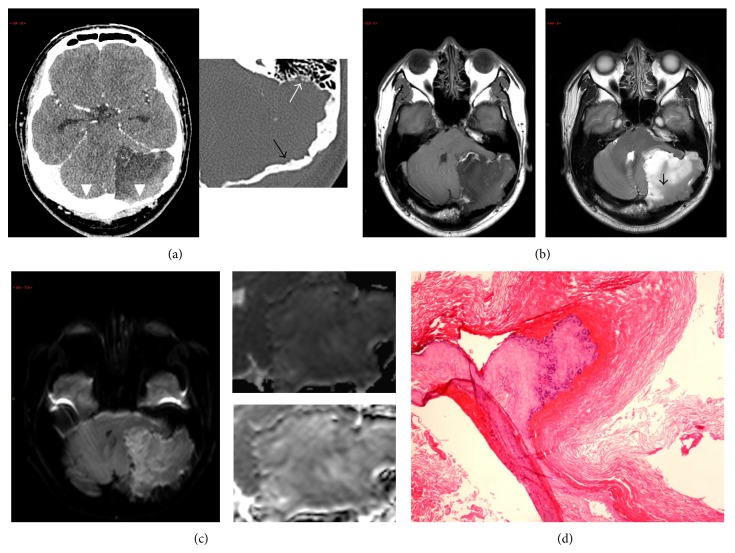
(a) Plain CT brain (left) and magnified bone window (right) show an irregular, infiltrative mass in the left posterior cranial fossa with invasion into the cerebellum, calvarium (black arrow), and mastoid antrum (white arrow). The transverse sinus (white arrowhead) has also been invaded (compared to the normal transverse sinus on the right). No appreciable contrast enhancement is identified after IV contrast (not shown). Note the lack of perifocal oedema despite the aforementioned aggressive features. (b) T1-weighted (left) and T2-weighted (right) MRI images of the epidermoid cyst show the typical T1-hypo- and T2-hyperintense signal of an epidermoid cyst in the more anterior component. Heterogeneous signal intensity is noted in the most posterolateral component (black arrow). There is no perilesional oedema or appreciable contrast enhancement after IV gadolinium (not shown). (c) DWI with *b* factor 1000 (left panel), masked ADC (right upper), and unmasked ADC (right lower image) demonstrate no significant restricted diffusion, as evident by the grossly isointense signal seen in the DWI and raw unmasked ADC map. (d) H&E section (×100 magnification) of the postsurgical specimen shows thick cyst wall of keratinizing squamous epithelium and amorphous keratin. Findings are compatible with an epidermoid cyst.

A 37-year-old male with good past health was admitted for progressive unsteady gait for six months. CT brain (Figure 2(a)) showed an extra-axial hypodense lesion with cystic and calcific components in the left posterior cranial fossa with invasion into skull vault, meninges, cerebellum, and the cerebral sinuses. MRI brain (Figure 2(b)) demonstrated heterogeneous T1W and hyperintense T2W signals, with no restricted diffusion (Figure 2(c)). Gross total excision was performed, and the patient recovered after complicated and postoperative rhinorrhea. Histology of surgical specimen demonstrated cyst capsule made of mature keratinizing squamous epithelium (Figure 2(d)), with flakes of keratin and dystrophic calcification, again compatible with an epidermoid cyst.

## 4. Discussion

Epidermoid cysts can occur throughout the neuroaxis, most commonly in the cerebellopontine angles (40–50%) and the parasellar region [1]. Conversely, atypical epidermoid cysts are rare, with intra-axial epidermoid cysts accounting for less than 1.5% of all intracranial epidermoid cysts [3] and intradiploic epidermoid cysts (including congenital cholesteatomas) accounting for ~3% [4, 5]. Previous case reports have found that 80% of reported intraparenchymal epidermoid cysts involve the frontal and temporal lobes [3] and occasionally the pineal gland [6] or the brainstem [7]. The proposed embryological pathogenesis of the typical epidermoid cyst involves trapped ectodermal components travelling along the otic vesicles during neural tube closure, thus accounting for the propensity for its location at the cerebellopontine angles [8]. Along this line of reasoning, intraparenchymal epidermoids are thought to arise when the ectodermal inclusion occurs before the third week of embryogenesis (when the primary cerebral vesicle is being formed), while intradiploic epidermoid cysts originate from aberrant ectodermal remnants that become trapped after neural tube closure [3, 4, 8].

Computed tomographic features for the typical epidermoid cysts include a hyperdense lobulated mass without contrast enhancement [9, 10], a finding we observed in patient 1 and the parts of the lesion in patient 2. This hyperdensity stems from a combination of proteinaceous content, saponification of keratinized debris, leukocytes, and lipid debris [1]. We observed T1 hyper- and marked T2 hypointense signals in patient 1, a pattern that was opposite to that of the typical epidermoid cyst (T1 hypo- and T2 hyperintensity). This difference is best explained with the principle that T1- and T2-weighted MRI signals are heavily influenced by protein content. More specifically, in a sinonasal secretion study [11] in which the authors studied the effects of protein content on MRI signal intensity, it was found that bright T1 and extremely dark T2 signals (a pattern that best described our case 1 lesion) were a result of at least 30% of protein content. This corresponded to the intraoperative finding for which the lesion was so viscous that it was not amenable to excision but required removal by a scoop. This high protein content (which resulted in extreme T2 hypointensity) also accounted for the lack of restricted diffusion, in which DWI are actually T2-weighted images made sensitive to diffusion by strong gradient pulses [11]. Note is made that the corresponding ADC (Figure 1(c)) appeared darkened (which could be interpreted as a sign of restricted diffusion); it was a result of postprocessing of “masking” which ignored very low signal intensity in an image. We were able to “unmask” the ADC images of the original raw data (Figure 1(c)) which confirmed the lack of restricted diffusion. This “protein-dependent T2 signal” explanation could only partly account for the lack of restricted diffusion in our case 2 (only slight hypointensity on T2W), and there must be an additional (yet unknown) factor accounting for its imaging feature [9, 10]. While there is a lack of unifying imaging features in atypical epidermoid cysts, our findings at least suggested that the intraparenchymal or intradiploic epidermoid cysts can present with atypical T1W and T2W signal and no restricted diffusion. In addition, while the typical epidermoid cysts are often described as “soft lesions” conforming to or insinuating between brain structures [1], the invasive nature of the second case suggests that atypical epidermoid cysts can behave aggressively with invasion into surrounding structures.

The radiological differential diagnoses of an epidermoid cyst include an arachnoid cyst, dermoid cyst, abscesses, metastasis, or slow growing brain tumours. FLAIR and DWI sequences [12] have been proposed as useful discriminator in differentiating an arachnoid cyst from the typical epidermoid cyst, in which the signal intensity of the arachnoid cyst follows CSF signal without restricted diffusion. A dermoid cyst is usually at a more central location with foci of calcifications which could be differentiated from epidermoid cyst [13]. Abscesses show typical rim of the contrast enhancement, metastasis is usually multiple with known primary tumour and heterogeneous contrast enhancement, and cystic neoplasm usually shows solid component with enhancement. Thus, in spite of the atypical MRI signals seen in our two cases, the combination of hyperdense cyst, the lack of contrast enhancement, and surrounding edema is a useful clue in raising epidermoid cyst as a diagnosis.

Current debate is ongoing regarding the optimal treatment for an epidermoid cyst. On the one hand, gross total resection of epidermoid cysts offers a definite treatment with prevention of its occurrence or aseptic meningitis [5]. Conversely, epidermoids are often located in close proximity to neurovasculature and vital brain parenchyma, and thus a conservative resection can be considered given the slow growing nature of this tumour, with a linear growth rate similar to normal epithelial cells (~one generation per month) [14]. Our second case illustrated that, with its invasiveness into meninges, skull vault, and cerebral sinuses, total excision presents as a definite surgical challenge and increases postoperative complications such as CSF leakage.

## 5. Conclusion

Epidermoids outside the typical location are exceedingly rare lesions with atypical imaging features, including reversal of the typical T1 and T2 signal, as well as a lack of restricted diffusion. In spite of their slow growth, they also can present with adjacent structure invasion that requires meticulous neurosurgical intervention.

## References

[1] Osborn A. G., Preece M. T. (2006). Intracranial cysts: radiologic-pathologic correlation and imaging approach. *Radiology*.

[2] Kurosaki K., Hayashi N., Hamada H., Hori E., Kurimoto M., Endo S. (2005). Multiple epidermoid cysts located in the pineal and extracranial regions treated by neuroendoscopy—case report. *Neurologia Medico-Chirurgica*.

[3] Aribandi M., Wilson N. J. (2008). CT and MR imaging features of intracerebral epidermoid—a rare lesion. *The British Journal of Radiology*.

[4] Ichimura S., Hayashi T., Yazaki T., Yoshida K., Kawase T. (2008). Dumbbell-shaped intradiploic epidermoid cyst involving the dura mater and cerebellum. *Neurologia Medico-Chirurgica*.

[5] Khan A. N., Khalid S., Enam S. A. (2011). Intradiploic epidermoid cyst overlying the torcula: a surgical challenge. *BMJ Case Reports*.

[6] MacKay C. I., Baeesa S. S., Ventureyra E. C. G. (1999). Epidermoid cysts of the pineal region. *Child's Nervous System*.

[7] Sari A., Ozdemir O., Kosucu P., Ahmetoglu A. (2005). Intra-axial epidermoid cysts of the brainstem. *Journal of Neuroradiology*.

[8] Berhouma M., Bahri K., Jemel H., Khaldi M. (2006). Intracerebral epidermoid tumor: pathogenesis of intraparenchymal location and magnetic resonance imaging findings. *Journal of Neuroradiology*.

[9] Kallmes D. F., Provenzale J. M., Cloft H. J., McClendon R. E. (1997). Typical and atypical MR imaging features of intracranial epidermoid tumors. *The American Journal of Roentgenology*.

[10] Som P. M., Dillon W. P., Fullerton G. D., Zimmerman R. A., Rajagopalan B., Marom Z. (1989). Chronically obstructed sinonasal secretions: observations on T1 and T2 shortening. *Radiology*.

[11] le Bihan D., Poupon C., Amadon A., Lethimonnier F. (2006). Artifacts and pitfalls in diffusion MRI. *Journal of Magnetic Resonance Imaging*.

[12] Hakyemez B., Aksoy U., Yildiz H., Ergin N. (2005). Intracranial epidermoid cysts: diffusion-weighted, FLAIR and conventional MR findings. *European Journal of Radiology*.

[13] Smirniotopoulos J. G., Chiechi M. V. (1995). Teratomas, dermoids, and epidermoids of the head and neck. *Radiographics*.

[14] Collins V. P., Loeffler R. K., Tivey H. (1956). Observations on growth rates of human tumors. *The American Journal of Roentgenology, Radium Therapy, and Nuclear Medicine*.

